# Transcriptomic and metabolomic analyses reveals keys genes and metabolic pathways in tea (*Camellia sinensis*) against six-spotted spider mite (*Eotetranychus Sexmaculatus*)

**DOI:** 10.1186/s12870-023-04651-8

**Published:** 2023-12-11

**Authors:** Xiaoping Wang, Yunjia Xiang, Minshan Sun, Yuanyuan Xiong, Chunhua Li, Ting Zhang, Weiwei Ma, Yun Wang, Xiao Liu

**Affiliations:** 1 Tea Refining and Innovation Key Laboratory of Sichuan Province, Tea Research Institute, Sichuan Academy of Agricultural Sciences, Chengdu, China; 2 Institute of Plant Protection, Sichuan Academy of Agricultural Sciences, Chengdu, China; 3 Henan Assist Research Biotechnology Co., Ltd, Zhengzhou, China

**Keywords:** Tea plant, Spider mite, Transcriptome, Metabolome, Plant–insect interaction

## Abstract

**Background:**

Six-spotted spider mite (*Eotetranychus sexmaculatus*) is one of the most damaging pests of tea (*Camellia sinensis*). *E. sexmaculatus* causes great economic loss and affects tea quality adversely. In response to pests, such as spider mites, tea plants have evolved resistance mechanisms, such as expression of defense-related genes and defense-related metabolites.

**Results:**

To evaluate the biochemical and molecular mechanisms of resistance in *C. sinensis* against spider mites, “*Tianfu-5*” (resistant to *E*. *sexmaculatus*) and “*Fuding Dabai*” (susceptible to *E*. *sexmaculatus*) were inoculated with spider mites. Transcriptomics and metabolomics based on RNA-Seq and liquid chromatography coupled to tandem mass spectrometry (LC-MS/MS) technology were used to analyze changes in gene expression and metabolite content, respectively.

RNA-Seq data analysis revealed that 246 to 3,986 differentially expressed genes (DEGs) were identified in multiple compared groups, and these DEGs were significantly enriched in various pathways, such as phenylpropanoid and flavonoid biosynthesis, plant–pathogen interactions, MAPK signaling, and plant hormone signaling. Additionally, the metabolome data detected 2,220 metabolites, with 194 to 260 differentially abundant metabolites (DAMs) identified in multiple compared groups, including phenylalanine, lignin, salicylic acid, and jasmonic acid. The combined analysis of RNA-Seq and metabolomic data indicated that phenylpropanoid and flavonoid biosynthesis, MAPK signaling, and Ca2+-mediated PR-1 signaling pathways may contribute to spider mite resistance.

**Conclusions:**

Our findings provide insights for identifying insect-induced genes and metabolites and form a basis for studies on mechanisms of host defense against spider mites in C. sinensis. The candidate genes and metabolites identified will be a valuable resource for tea breeding in response to biotic stress.

**Supplementary Information:**

The online version contains supplementary material available at 10.1186/s12870-023-04651-8.

## Introduction

Tea (*Camellia sinensis*) has been used in China as both a beverage and medicine for centuries, and its popularity spread worldwide during the Tang Dynasty along the Silk Road [1]. Tea has become one of the most widely consumed beverages worldwide; therefore, tea plant cultivation has expanded globally [1]. However, tea plants face significant challenges from approximately 1,000 arthropod species that can harm root, stem, leaf, flower, seed of the tea plant, resulting in yield losses of up to 55% [2]. Spider mites are the most detrimental and persistent pests in tea production [3]. Spider mites often lacerate the cells, causing minute reddish-brown marks on the upper surface of mature leaves, yielding up to 46% crop loss [2].

Effectively controlling spider mites is crucial for various aspects of tea cultivation, such as management, breeding, yield, and income generation. *E. sexmaculatus* is a spider mite of the family Tetranychidae, originally from North America, has become a major pest in Chinese tea production [4]. However, the controlling of six-spotted spider mite are scarcely reported. Although pesticides are effective on a wide range of pests, their use increases environmental pollution, increases accumulation of pesticide residue, and promotes pest resistance [2, 5–7].

Plants have an innate defense mechanism against biotic and abiotic stress. When faced with herbivorous insects, plants frequently regulate or induce changes in defense-related genes, chemicals, biochemistry, and morphology to develop a comprehensive defense strategy [8]. This provides ideas for screening and breeding varieties with natural pest resistance properties, to address environmental concerns.

Although there are several reports on host resistance in *C. sinensis*, few resistance genes and metabolites are known, because studies on breeding for insect resistance started late in *C. sinensis*. Tea plants with higher rhodoxanthin content are more susceptible to *Oligonychus coffeae* [9], and highly resistant and susceptible *C. sinensis* lines have been screened based on differences in resistance [10]. Volatile beta-ocimene, higher catechins, phenylalanine ammonia lyase, and glutamate dehydrogenase increase resistance of *C. sinensis* against *O. coffeae* [11, 12]. Studies on other higher plants have suggested the involvement of calcium ion signaling, phosphorylation cascades, leucine-rich repeat receptor-like kinases, and salicylic acid and jasmonic acid pathways [13, 14]. Because resistance genes in tea have been rarely reported, selection and use of resources for breeding insect resistant tea plants is limited.

High-throughput transcriptomic and metabolite identification technologies have been extensively used to study plant–insect interactions [15–21]. These techniques provide a basis for the rapid access of candidate insect resistance genes and metabolites in tea plants. In this study, the “*Tianfu-5”* (resistant to *Eotetranychus sexmaculatus*, R) and “*Fuding Dabai*” (susceptible to *E*. *sexmaculatus*, S) varieties were used. RNA-Seq and metabolomic analysis were utilized to identify candidate genes and metabolites. The results indicated that these genes and metabolites may involve in spider mite resistance. The significant enrichment pathways of these DEGs and DAMs include phenylpropanoid biosynthesis, salicylic acid, jasmonic acid, and plant–pathogen interaction pathways, which may play a role in regulating resistance gene expression. The results provide valuable information for studies on gene expression in response to arthropods in tea, as well as molecular breeding of tea.

## Materials and methods

### Plant materials

We used two varieties of *C*. *sinensis*: “*Tianfu-5*” (resistant to *E*. *sexmaculatus*, R) and “*Fuding Dabai*” (susceptible to *E*. *sexmaculatus*, S). The plants were cultivated under the same soil and environmental conditions in Meishan City, Sichuan province (29.81°N, 103.17°E). Resistance to *E*. *sexmaculatus* was independently investigated every yr for 3 yrs (2017–2019). Two-yr-old tea plants (cutting) grown under a 16-h light (25 °C)/8-h dark (20 °C) photoperiod in the greenhouse were used for insect feeding treatment. *E*. *sexmaculatus* was collected from the organic tea garden in Yucheng District (Ya’an, Sichuan) and bred with young leaves of *Fuding Dabai* at 26 °C ± 1 °C and relative humidity 80%. Then 50 *E*. *sexmaculatus* larvae were distributed evenly on new shoots of tea plants. The base of leaf’s petiole was covered with cotton wool impregnated with glycerol to prevent the migration of spider mites between leaves. Subsequently, *E*. *sexmaculatus* was removed and tea leaves were collected at three time points: non-feeding (d 0), 2-d post-feeding, and 8-d post-feeding. Three and six biological replicates were harvested from each group for RNA-Seq and metabolomic analyses, respectively. Leaves of *Tianfu-5* (R) were labeled R0, R2, and R8 and those of *Fuding Dabai* (S) were labeled S0, S2, and S8 for d 0, d 2, and d 8, respectively. All samples were frozen in liquid nitrogen and stored at − 80 °C until use.

### Resistance index assays

The resistance index of varieties R and S was assayed in the field in April–May every yr. Three leaves on a new shoot were randomly selected from ten tea plants, and the number of *E*. *sexmaculatus* on each leaf was counted. Disease grading standards were grade 0: no spider mite, grade 1: 1 ≤ number of spider mites ≤ 5, grade 2: 6 ≤ number of spider mites ≤ 20, grade 3: 21 ≤ number of spider mites ≤ 50, and grade 4: 51 ≤ number of spider mites. Disease index (DI) was used to calculate resistance using the following formula:

DI = Σ (number of each grade leaves × representative value of this level) / (total number of leaves × 4) × 100. Resistance criteria were DI ≤ 5: resistant, 5 < DI ≤ 15: mildly resistant, 15 < DI ≤ 25: susceptible, and DI > 25: highly susceptible.

### RNA extraction, library construction, and sequencing

Similar-sized leaves from both varieties were harvested at predetermined time points, washed with deionized water, frozen in liquid nitrogen, and stored at − 80 °C for RNA extraction. Total RNA was extracted from root tissue using TRIzol reagent (Invitrogen, Carlsbad, CA, USA). mRNA was enriched using oligo(dT) beads, fragmented using fragmentation buffer, and reverse transcribed into cDNA using NEBNext Ultra RNA Library Prep Kit for Illumina (NEB #7530, New England Biolabs, Ipswich, MA, USA). The cDNA fragments were purified and ligated using Illumina sequencing adapters. Ligation products were selected based on size distribution using agarose gel electrophoresis and PCR amplified. The resulting cDNA library was sequenced using Illumina NovaSeq 6000.

### RNA-Seq data analysis

Adapter sequences, as well as low-quality reads, were removed from each dataset using fastp (version 0.18.0) [22]. The short read alignment tool Bowtie2 [23] (version 2.2.8) was used for mapping reads to the rRNA database, and rRNA-mapped reads were removed. Clean reads were used for assembly and calculation of gene abundance. Reference *C*. *sinensis* genome and annotation files were downloaded from the BIG database (accession no. GWHACFB00000000; https://bigd.big.ac.cn/search/?dbId=gwh&q=GWHACFB00000000) [24]. An index of the reference genome was built, and paired-end clean reads were mapped to the reference genome using HISAT2 (version 2.2.4) with “–rna-strandness RF” and other parameters set as default [25]. The reconstruction of transcripts was performed using StringTie (version 1.3.1) and HISAT2 [25, 26].

For each transcription region, fragments per kilobase of transcript per million mapped reads (FPKM) values were calculated to quantify expression abundance and variations using RSEM software [27]. Differentially expressed gene (DEG) analysis was performed with DESeq2 [28]. Transcripts with absolute value of log2(fold change) ≥ 1 and false discovery rate (FDR) ≤ 0.05 were considered differentially expressed.

### Metabolite extraction

In total, 100 mg tissue was ground with liquid nitrogen, resuspended in prechilled 80% methanol and 0.1% formic acid by vortexing, incubated on ice for 5 min, and centrifuged at 15,000 × *g* and 4 °C for 20 min. The supernatant was diluted with liquid chromatography mass spectrometry (LC-MS) grade water to achieve a final concentration of 53% methanol. The samples were transferred to a fresh Eppendorf tube and centrifuged at 15,000 × *g* and 4 °C for 20 min. The supernatant was injected into a liquid chromatography with tandem mass spectrometry (LC-MS/MS) system [29]. Quality control samples included equal volumes from each experimental sample.

### UHPLC-MS/MS analysis

UHPLC-MS/MS analysis was performed using Vanquish UHPLC System (Thermo Fisher, Germany) coupled with an Orbitrap Q ExactiveTM HF-X mass spectrometer (Thermo Fisher, Germany). Samples were injected onto a Hypesil Gold column (100 × 2.1 mm, 1.9 μm) (Thermo Fisher, USA) using a 17-min linear gradient at flow-rate 0.2 mL/min. For the positive polarity mode, eluents A (0.1% FA in water) and B (methanol) were used; for the negative polarity mode, eluents A (5 mM ammonium acetate, pH 9.0) and B (methanol) were used. The solvent gradient was set as follows: 2% B, 1.5 min; 2–100% B, 12.0 min; 100% B, 14.0 min; 100–2% B, 14.1 min; 2% B, 17 min. Q ExactiveTM HF-X mass spectrometer was operated in positive/negative polarity mode with spray voltage 3.2 kV, capillary temperature 320 °C, sheath gas flow-rate 40 arb, and aux gas flow-rate 10 arb.

### Data processing and metabolite identification

Raw data files generated by UHPLC-MS/MS were processed using Compound Discoverer 3.1 (CD3.1, Thermo Fisher) to perform peak alignment, peak picking, and quantitation of each metabolite. The main parameters were set as follows: retention time tolerance, 0.2 min; actual mass tolerance, 5 ppm; signal intensity tolerance, 30%; signal-to-noise ratio, 3; and minimum intensity, 100,000. Raw data were normalized to predict a molecular formula based on additive ions, molecular ion peaks, and fragment ions. Peaks were matched using the mzCloud (https://www.mzcloud.org/), mzVault, and Mass List databases to obtain qualitative and relative quantitative results. PCA analysis was performed using the gmodels package in R (version 2.18.1). To screen significant differentially abundant metabolites (DAMs) between different comparison groups, we combined variable importance in projection (VIP) values of the multivariate statistical analysis orthogonal projection to latent structures-discriminant analysis (OPLS-DA) and *t*-test *P*-values obtained from univariate statistical analysis, using the ropls package in R (version: 1.31.0) [30–32]. The criteria to screen significantly changed metabolites were VIP ≥ 1 and *t*-test *P* value < 0.05 in the OPLS-DA model [33]. Metabolite Set Enrichment Analysis was performed using the MetaboAnalyst package in R (version: 4.0) [34].

### Functional enrichment of DEGs and DAMs

The expression patterns of genes and metabolites were analyzed using short time-series expression miner (STEM) (version 1.3.8.43) [35]. DEGs or DAMs were subjected to enrichment analysis of gene ontology (GO) functions and Kyoto Encyclopedia of Genes and Genomes (KEGG) pathways using GO Term Finder and PathFinder, respectively [36, 37]. *P*-values for each treatment stage were calculated using FDR correction, considering FDR ≤ 0.05 as threshold. For each KEGG pathway, the number of up- and downregulated genes of a variety was compared with that of the reference set using Fisher’s exact test to identify enriched pathways.

### Correlation analysis of RNA-Seq and metabolomic data

We performed correlation analysis of RNA-Seq data and metabolomic data to examine the correlation between DEGs and DAMs. First, we queried the KEGG metabolic pathways shared by genes and metabolites, and analyzed the associated characteristics between genes and metabolites in the shared pathways. Second, a two-way orthogonal projections to latent structures (O2PLS) model was constructed using gene expression and metabolite abundance data with the OmicsPLS package in R [38]. Third, Pearson’s correlation coefficients between gene expression and metabolite abundance were calculated [39].

## Results

### Resistance of tea varieties *Tianfu-5*  and *Fuding Dabai* to the spider mite

**Fig. 1:**
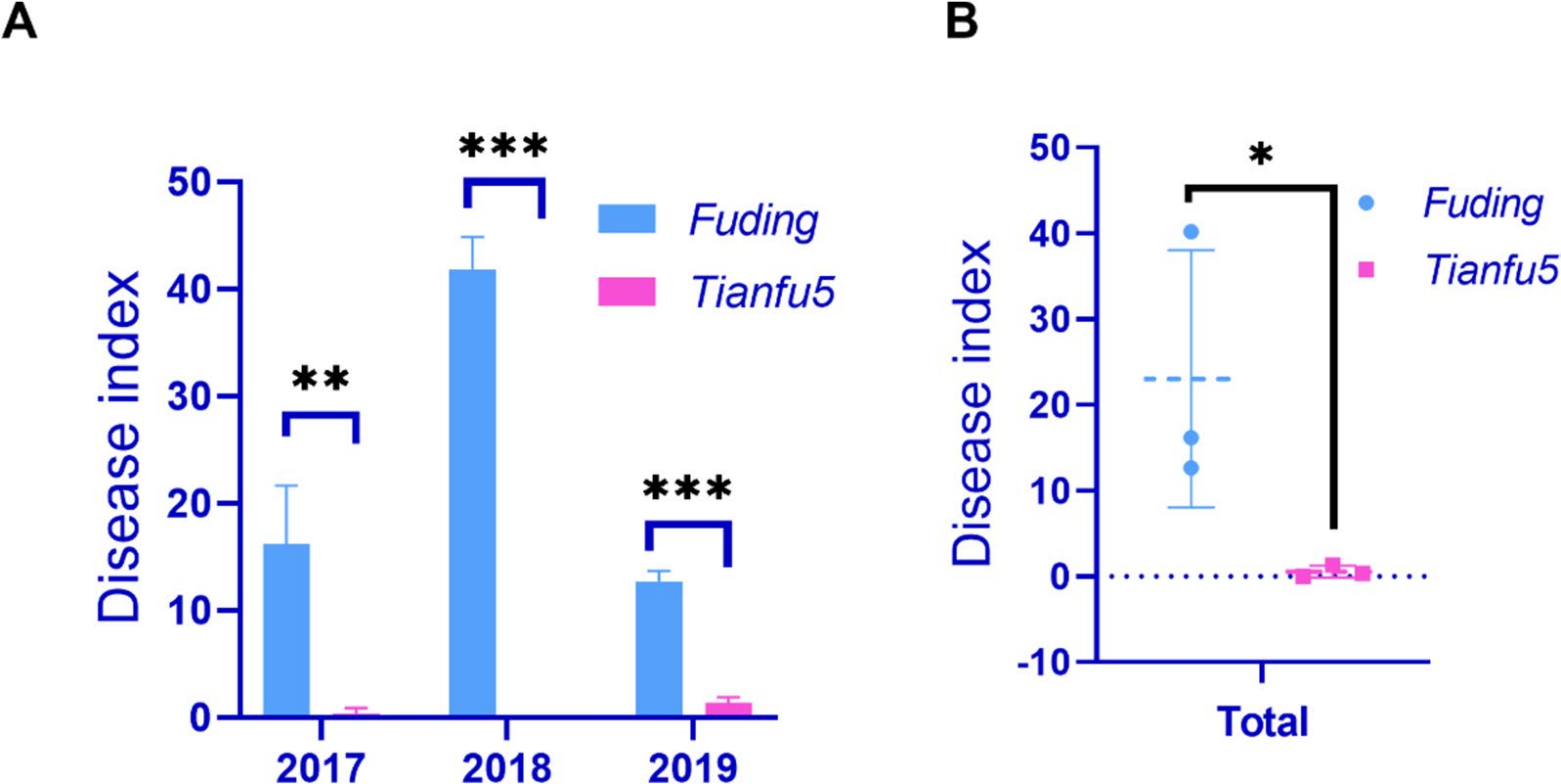
The resistance of two tea plant varieties to spider mite. **A** Disease index form 2017–2019. **B** the total disease index. Error bars indicate the SD (n ≥ 3) of three biological replicates. Asterisks indicate statistically significant differences as determined by Student’s t-test (*P < 0.05, **P < 0.01, ***P < 0.001)

The resistance of *C*. *sinesis* varieties *Tianfu-5* (R) and *Fuding Dabai* (S) against spider mites was investigated from 2017 to 2019. The DI value of *Fuding Dabai* was significantly higher than that of *Tianfu-5* (Fig. 1A). However, in the spring of 2018, the climate was characterized by drought and high temperatures, potentially leading to a large variation range for the DI of *Fuding Dabai* in this year. The statistical results imply that the impact of temperature and drought on the DI of susceptible varieties may be greater than that of resistant varieties (Fig. 1B).

### RNA-Seq data

Totally, 18 RNA-Seq libraries (six samples with three biological replicates) were constructed, and 4.5 GB raw data were obtained per library. Low-quality reads were filtered to obtain 99.6% raw reads per library, defined as clean reads (Supplementary Table 1). We obtained > 94.1% of Q30 values and > 44.1% of GC content (Supplementary Table 2). Clean reads were mapped to the tea reference genome. Approximately 88% of the total reads (including 85% unique and 3.5% multiple reads) were mapped to the genome (Supplementary Table 3). Of these, approximately 73% were mapped to exons, 13.5% to introns, and 13.5% to intergenic regions (Supplementary Table 4). Following transcript mapping and reconstruction, 33,556 known and 5,228 novel genes were obtained to compute FPKM read values of each sample.

### Analysis of DEGs

**Fig. 2:**
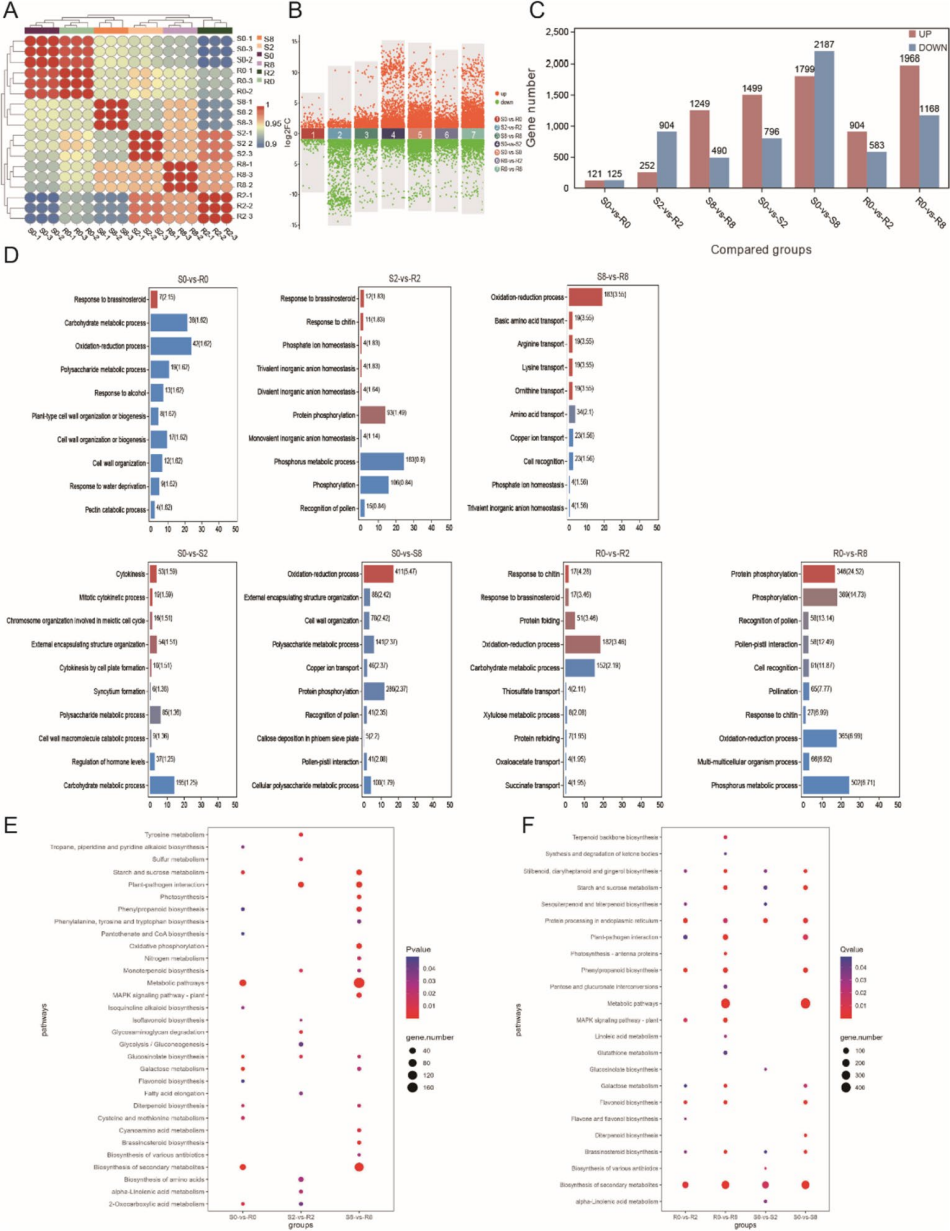
Analysis of DEGs. **A** Correlation coefficients between gene expression datasets. Red and blue colors indicate positive and negative correlation coefficients between samples, respectively. **B** Scatter plot of DEGs. Red and green colors indicate up-regulated genes and down-regulated genes in multiple compared groups. **C** Number of DEGs among compared groups. **D** GO analysis of seven compared groups. Red and blue colors indicate significant enrichment (-log10(Qvalue)). Enrichment gene number is shown behind the bars, and the numbers in brackets indicate -log10(Pvalue). **E** KEGG analysis of DEGs between S and R at three time points. **F** KEGG analysis of DEGs after feeding spider mite for 2 days and 8 days. The color and size of the bubbles indicate significant enrichment and gene number, respectively

Correlation analysis assesses repeatability of samples and degree of variation between samples. Correlation analysis of 18 samples showed values > 0.9. Values for all three samples at each time point were highly reproducible. Correlation between S0 and R0 was highest among samples, suggesting that although the two samples differed greatly in resistance, differences in their genetic backgrounds were lower than changes in genetic differences resulting due to spider mite feeding (Fig. 2A). Therefore, we focused on differences between varieties and changes in differences between time points.

Therefore, seven comparison groups were set up. The results showed 246 DEGs (121 up- and 125 downregulated genes) between the two tea plant varieties (S0 and R0) before spider mite feeding, indicating that the genetic backgrounds of the two varieties varied. Two days after mite feeding, the number of DEGs between S2 and R2 increased to 1,156 DEGs (252 up- and 904 downregulated genes). After 8-d feeding, the number of DEGs between S8 and R8 increased to 1,739 DEGs (1,249 up- and 490 downregulated genes) (Fig. 2B, C). Next, we compared gene expression levels over time. For variety S, we found 2,295 DEGs in group S0-vs-S2 (1,499 up- and 796 downregulated genes) and 3,986 DEGs in group S0-vs-S8 (1,799 up- and 2,187 downregulated genes). For variety R, we found 1,487 DEGs in group R0-vs-R2 (904 up- and 583 downregulated genes) and 3,136 DEGs in group R0-vs-R8 (1,968 up- and 1,168 downregulated genes) (Fig. 2B, C).

GO enrichment analysis was used to evaluate the function of the identified DEGs. Up- and downregulated DEGs in varieties R and S after spider mite feeding were enriched in biological process (BP), cellular component (CC), and molecular function (MF), including GO terms: cellular process, metabolic process, cell part, membrane part, and catalytic activity and binding (Supplementary Fig. 1). In group S0-vs-R0 (0-d feeding), GO terms response to brassinosteroid, etc., were significantly enriched. In group S2-vs-R2 (2-d feeding), GO terms response to chitin, protein phosphorylation, phosphate ion homeostasis, etc., were significantly enriched. In group S8-vs-R8 (8-d feeding), GO terms oxidation–reduction, basic amino acid transport, etc., were significantly enriched (Fig. 2D).

We observed a significant difference in DEGs between varieties R and S after spider mite inoculation. In comparison groups S0-vs-S2 and S0-vs-S8, DEGs were not significantly enriched in response to stimulus-related GO terms. In groups R0-vs-R2 and R0-vs-R8, DEGs were significantly enriched in response to chitin. In group R0-vs-R2, DEGs were significantly enriched in response to brassinosteroid, oxidation–reduction processes, etc. In variety S plants, oxidation–reduction process was significantly enriched in S0-vs-S8 only, suggesting that earlier activation of oxidation–reduction pathways in variety R, as well as response to chitin and brassinosteroid, are important for resistance (Fig. 2D).

We performed KEGG enrichment analysis to evaluate DEG function in each comparison group. Significant enrichment was seen for genes involved in plant–pathogen interaction and monoterpenoid biosynthesis pathway in feeding comparison groups (S2-vs-R2 and S8-vs-R8). In S2-vs-R2, DEGs were significantly enriched in tyrosine metabolism, sulfur metabolism, isoquinoline alkaloid biosynthesis, indicating early resistance response (Fig. 2E).

In variety R, DEGs were significantly enriched in MAPK signaling and flavone and flavonol biosynthesis pathways. Two days after feeding, DEGs were significantly enriched in plant–pathogen interaction pathways in variety R. Comparatively, in variety S, enrichment occurred after 8-d feeding. Thus, the response time of the pathway including plant–pathogen interaction and phenylpropanoid biosynthesis was earlier in variety R than variety S (Fig. 2F).

### *C. sinensis* DEGs involved in response to spider mite

**Fig. 3:**
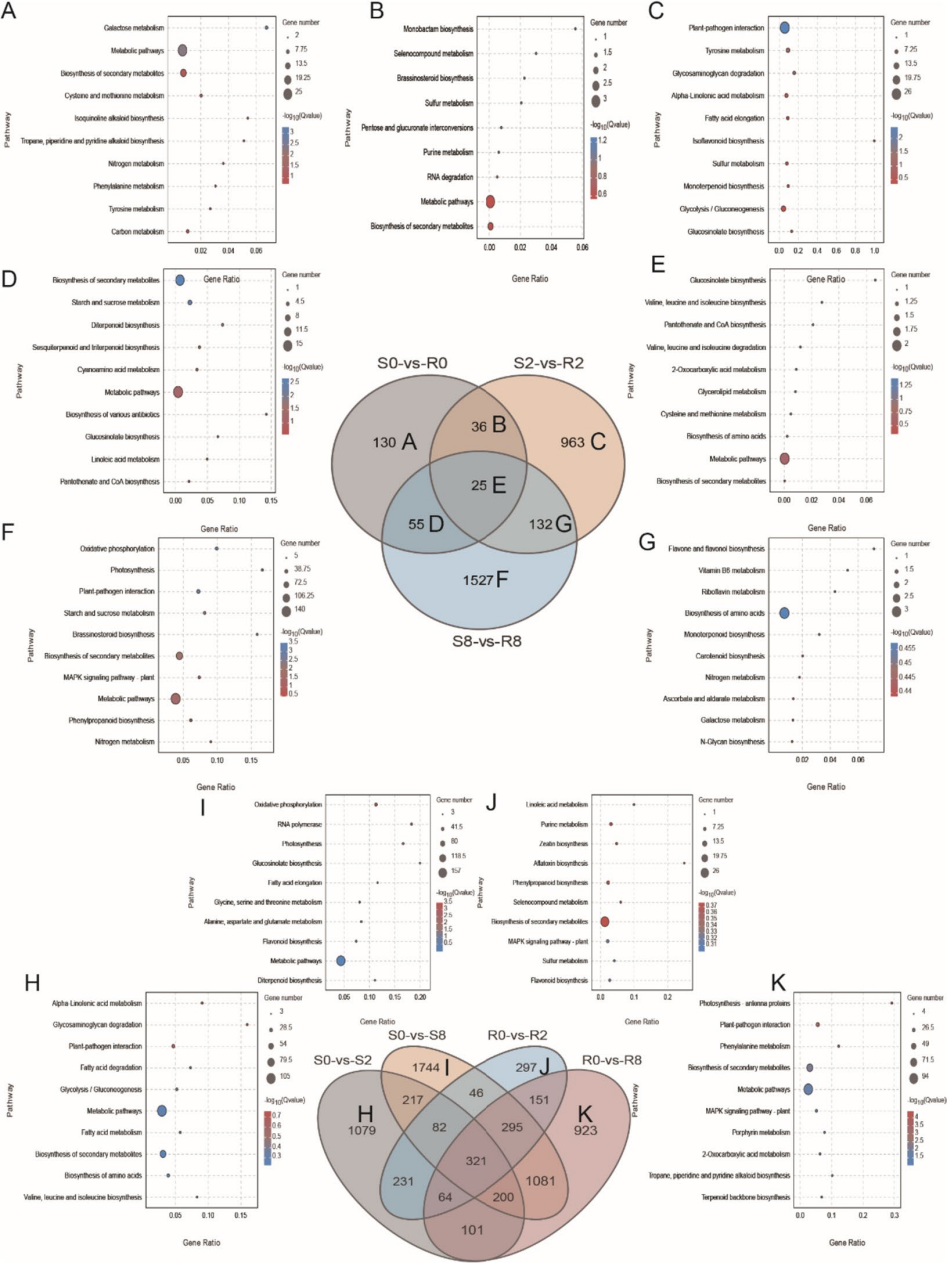
Venn diagram of the comparison groups and function enrichment analysis. **A-F** Lists of statistically significant DEGs between S and R at three time points both treatment and control were used to create a Venn diagram. **H-K** Lists of statistically significant DEGs between S and R after feeding spider mite for 2 days and 8 days were used to create a Venn diagram

Comparative analysis of DEGs between the two varieties showed that KEGG pathways corresponding to unique or shared DEGs at different times were enriched in metabolism (Fig. 3A–G). After 2-d feeding, significant enrichment was seen for plant–pathogen interaction, tyrosine metabolism, glycosaminoglycan degradation, isoflavonoid glycosaminoglycan degradation, isoflavonoid biosynthesis, monoterpenoid biosynthesis, etc. (Fig. 3C). These findings suggest similarities between stress response in *C. sinensis* to spider mite feeding and plant stress response to pathogenic bacteria. Enriched pathways of DEGs after 8-d feeding included oxidative phosphorylation, photosynthesis, plant–pathogen interaction, brassinosteroid biosynthesis, MAPK signaling pathway, phenylpropanoid biosynthesis, etc. (Fig. 3F), suggesting that prolonged leaf feeding not only affects the biotic stress response, but also photosynthesis at d 8.

Comparison of DEGs at different time points showed that in group S0-vs-S2, DEGs were enriched in alpha-linolenic acid metabolism, glycosaminoglycan degradation, plant–pathogen interaction, etc. (Fig. 3H). For group S0-vs-S8, DEGs were enriched in oxidative phosphorylation, etc. (Fig. 3I). For group R0-vs-R2, DEGs were enriched in linoleic acid metabolism, purine metabolism, phenylpropanoid biosynthesis, MAPK signaling, etc. (Fig. 3J). For group R0-vs-R8, DEGs were enriched in plant–pathogen interactions, phenylpropanoid metabolism, MAPK signaling pathway, etc. (Fig. 3K).

### Metabolome profiling of tea in response to spider mite feeding

**Fig. 4:**
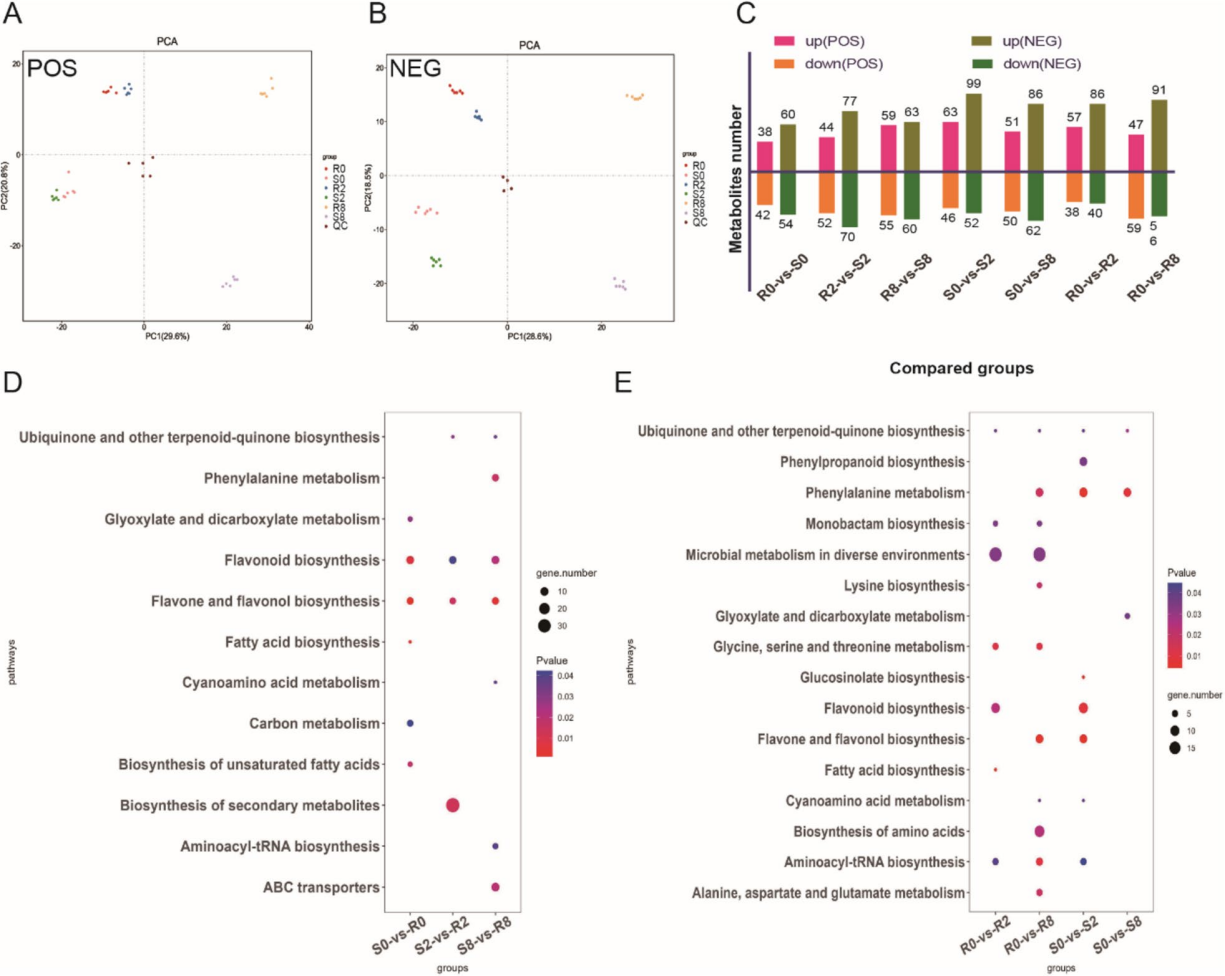
Different abundant metabolites (DAMs) of R and S tea plant fed by spider mite. **A** PCA plots of POS metabolism identified. **B** PCA plots of NEG metabolism identified. **C** Number of DAMs among compared groups. **D** KEGG analysis of DAMs between S and R at three time points. **E** KEGG analysis of DAMs after feeding spider mite for 2 days and 8 days

Investigation of metabolites produced by the two tea varieties in response to spider mite feeding showed that six replicates of each sample clustered both positive ion mode (POS) and negative ion mode (NEG) metabolites, whereas multiple samples differed significantly from each other (Fig. 4A, B). Through the identification of metabolites, we obtained 2,220 metabolites: 1,148 POS and 1,072 NEG (Supplementary Table 5).

DAMs were analyzed for the seven comparison groups based on transcriptome analysis. Comparison of DAMs at three timepoints revealed 80 POS and 114 NEG DAMs in group R0-vs-S0; 96 POS and 147 NEG DAMs in group R2-vs-S2; and 114 POS and 123 NEG DAMs in group R8-vs-S8. Comparison of DAMs at different timepoints in varieties R and S revealed 109 POS and 151 NEG DAMs in group S0-vs-S2; 101 POS and 148 NEG DAMs in group S0-vs-S8; 95 POS and 126 NEG DAMs in group R0-vs-R2; and 106 POS and 147 NEG DAMs in group R0-vs-R8 (Fig. 4C).

KEGG enrichment analysis revealed that DAM number was obviously less than DEG number. Although enrichment number and pathways between the two datasets were similar, significantly enriched pathway had large differences (Supplementary Fig. 2). After spider mite feeding (S2-vs-R2, S8-vs-R8), DAMs were significantly enriched pathways including: ubiquinone and other terpenoid–quinone biosynthesis, phenylalanine metabolism, ABC transporters, etc. (Fig. 4D). Comparison of differences between time points showed that the DAMs in variety R were significantly enriched in monobactam biosynthesis, microbial metabolism in diverse environments, glycine, serine, and threonine metabolism, and fatty acid biosynthesis (Fig. 4E).

### DAMs involved in the response of tea to the spider mite

**Fig. 5:**
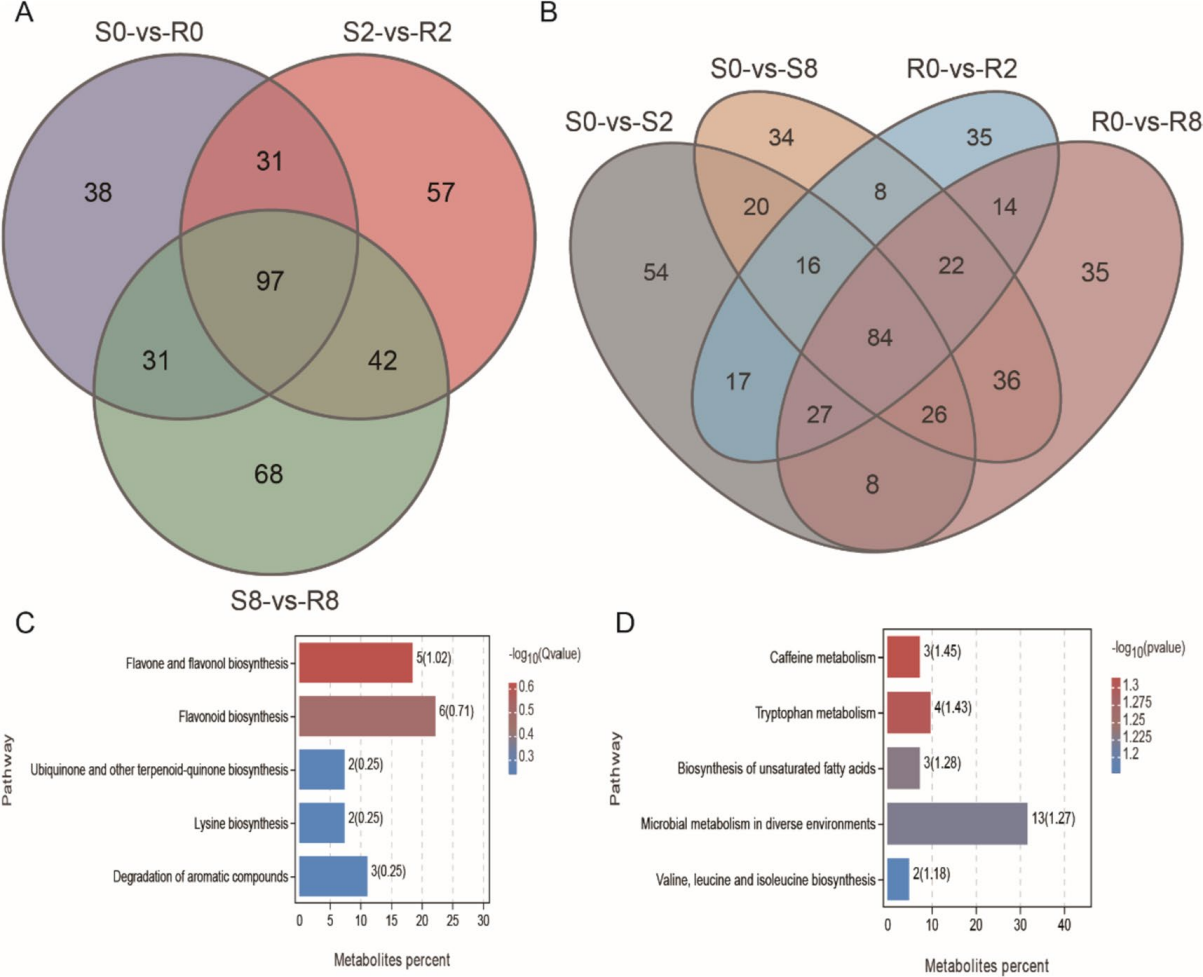
Venn diagram of the comparison groups and function enrichment analysis. **A** Lists of statistically significant DAMs between S and R at three time points both treatment and control were used to create a Venn diagram. **B** Lists of statistically significant DAMs between S and R after feeding spider mite for 2 days and 8 days were used to create a Venn diagram. **C** KEGG analysis of common DAMs between S and R at three time points. **D** KEGG analysis of DAMs only in R (R0-vs-R2, R0-vs-R8) or S (S0-vs-S2, S0-vs-S8) compared groups

The number of unique and common DAMs was lower than the number of DEGs in several comparison groups, suggesting that spider mites have a lower impact on metabolites than on gene expression (Fig. 5A, B). Functional enrichment analysis of the 97 DAMs common between both varieties at three time points, which may be related to constitutive differences in resistance between the varieties, showed that they were mainly associated with flavone and flavonol biosynthesis (Fig. 5C). DAMs specific for variety R (R0-vs-R2, R0-vs-R8) and S (S0-vs-S2, S0-vs-S8) may be related to different responses after spider mite feeding induction. KEGG enrichment analysis showed that these DAMs were mainly associated with caffeine metabolism, tryptophan metabolism, etc. (Fig. 5D).

### Combined RNA-Seq and metabolomic analysis

**Fig. 6:**
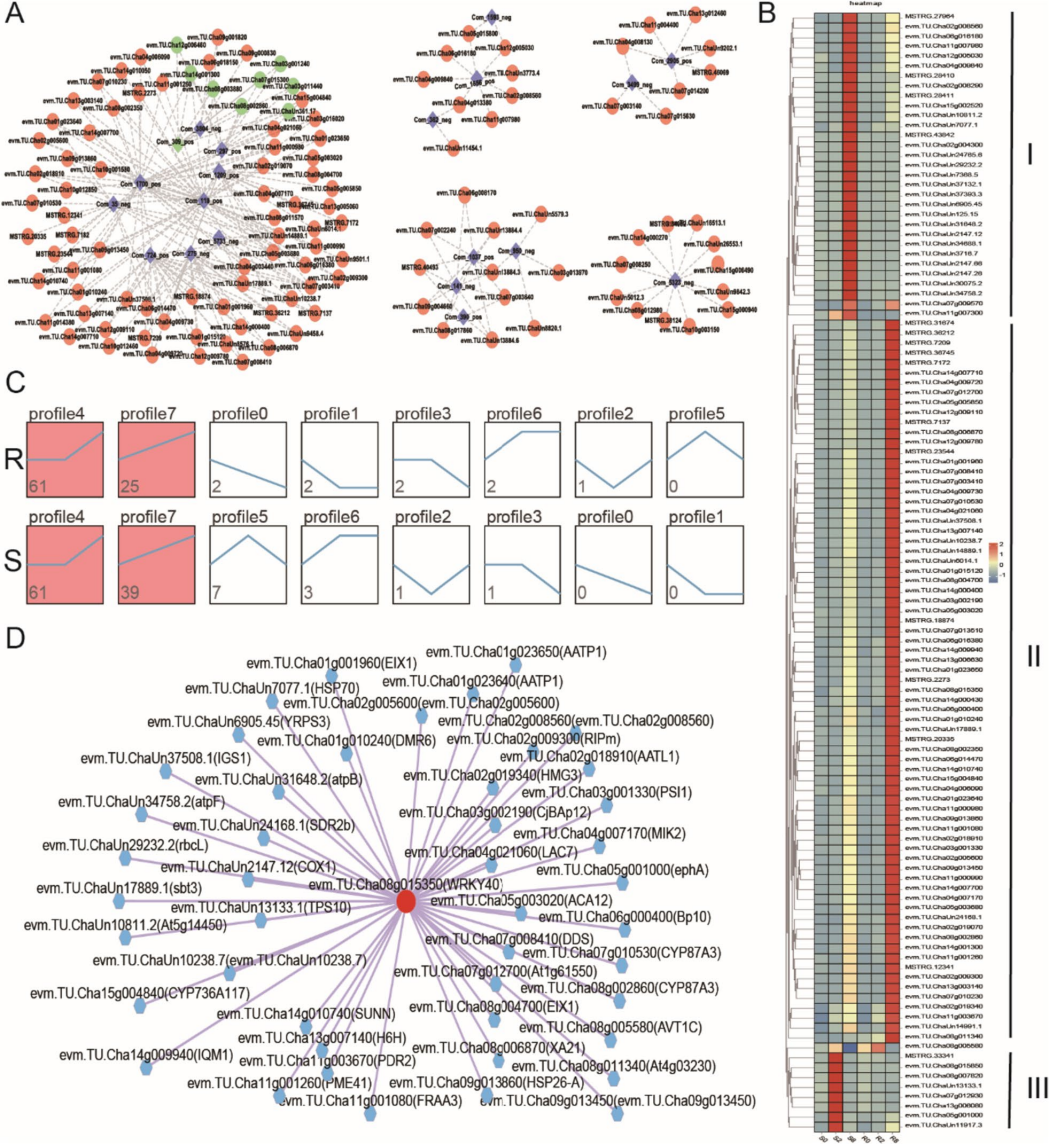
The connection network between DEGs and DAMs. **A** The connection network between DEGs and DAMs. The dots and diamonds represent genes and metabolites, respectively. Green color mean genes and metabolites associated with phenylpropanoid biosynthesis and flavonoid biosynthesis. **B** The expression pattern of 118 candidate DEGs. Three subgroups marked with cluster I, II, III. **C** The trend pattern analysis of DEGs expression level. Red color box meaning significant enrichment cluster. **D** The WRKY40 transcription factor target gene network

Pearson’s correlation coefficient was calculated for all DEGs and DAMs (Supplementary Table 6). Network diagrams were plotted for DEGs and DAMs with absolute Pearson’s correlation coefficient values > 0.5 and rank top 250. Using these criteria, we obtained five major networks, containing 23 DAMs and 118 DEGs, (Fig. 6A, Supplementary Fig. 3, Supplementary Table 7). Com_309_pos (2-(2-amino-3-methylbutanamido)-3-phenylpropanoic acid) is related to multiple DEGs, including evm.TU.Cha07g015300 (vinorine synthase-like), which is closely related to phenylpropanoid biosynthesis and flavonoid biosynthesis (Fig. 6A). Gene expression patterns divided the genes into three subgroups: (1) upregulated after 8-d feeding in variety S, no significant change in variety R (Cluster I) ; (2) weakly upregulated after 8-d feeding in variety S, significantly upregulated after 8-d feeding in variety R (Cluster II); (3) no significant change in variety R, significantly upregulated after 2-d feeding in variety S (Cluster III) (Fig. 6B). Expression analysis showed similar results, with both profile 4 and profile 7 patterns significantly enriched in varieties R and S; profile 4 genes significantly upregulated only after 8-d feeding; and profile 7 genes showing sustained increase after spider mite feeding (Fig. 6C). Further analysis showed that the WRKY40 transcription factor potentially targets 46 genes in > 118 DEGs (Fig. 6D, Supplementary Table 8).

### Phenylpropanoid biosynthesis pathways affect tea plant resistance to spider mites

**Fig. 7:**
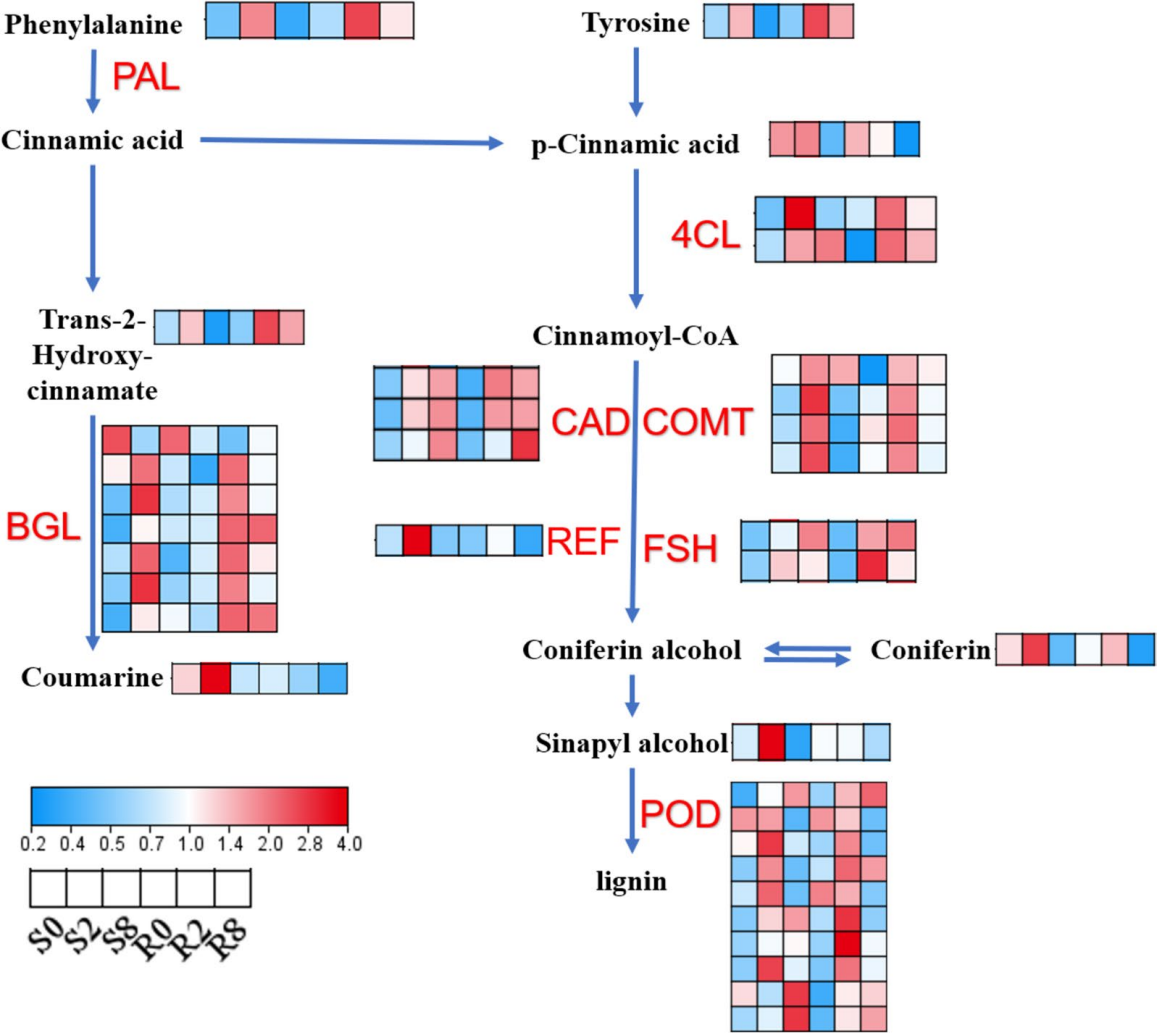
DEGs and DAMs in phenylpropanoid metabolic pathways. This figure has obtained the appropriate copyright permission to use the KEGG image. Supplementary Table 9 showed the FPKM value. Log2-scaled FPKM values are shown, ranging from low (blue) to high (red) expression. Within each box, rows represented different inoculation time points (from left to right). Red letter mean gene. Black letter showed metabolites. PAL, phenylalanine ammonia lyase. POD, peroxidase. BGL, betaglucosidase. 4CL, 4-coumarate–CoA ligase. CCoMT, caffeoyl-CoA O-methyltransferase. CAD, cinnamyl-alcohol dehydrogenase. REF, coniferyl-aldehyde dehydrogenase. FSH, ferulate-5-hydroxylase

Our findings revealed significant enrichment of phenylpropanoid biosynthesis pathways. DAM analysis revealed significant upregulation of several metabolites after spider mite feeding. Phenylalanine, tyrosine, and *trans*-2-hydroxy-cinamate were upregulated in variety R, whereas coumarin, coniferin, and sinapyl alcohol were upregulated in variety S. Combining changes of several genes in this pathway, it is evident that most of the genes exhibit upregulation in expression after 2 d of spider mite feeding. However, due to the integrated regulation of multiple homology betaglucosidase (*BGL*) and peroxidase (*POD*) genes, downstream metabolite content change may not align with the upstream metabolite trend (Fig. 7, Supplementary Table 9). This pathway is primarily associated with lignin and coumarin synthesis, suggesting that it is linked to spider mite resistance in tea.

### Salicylic acid and jasmonic acid are involved in tea plant resistance against spider mites

**Fig. 8:**
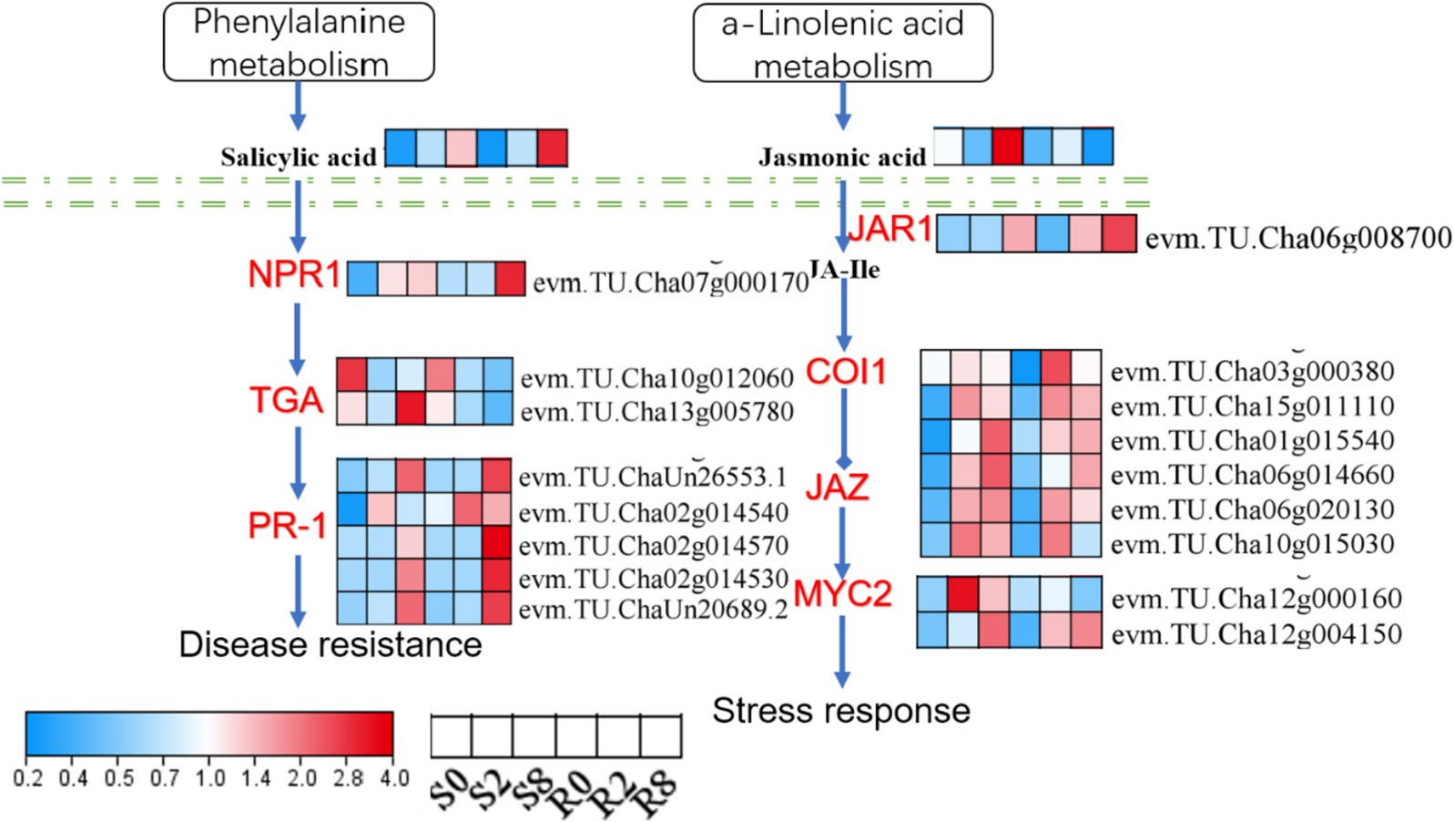
DEGs and DAMs in plant hormone signal transduction pathways. This figure has obtained the appropriate copyright permission to use the KEGG image. Log2-scaled fragments per kilobase per million (FPKM) values are shown, ranging from low (blue) to high (red) expression. Within each box, rows represented different inoculation time points (from left to right). Red letter mean gene. Black letter showed metabolites. NPR1, regulatory protein NPR1. TGA, transcription factor TGA. PR-1, pathogenesis-related protein 1. JAR1, jasmonic acid-amino synthetase. JAZ, jasmonate ZIM domain-containing protein. MYC2, transcription factor myelocytomatosis protein 2

Besides the phenylalanine metabolism pathway, linolenic acid metabolism was significantly enriched in several comparison groups. Both pathways have a direct regulatory effect on phytohormone production. After spider mite feeding, salicylic acid content gradually increased in varieties R and S. However, in variety S, jasmonic acid content decreased and then increased, whereas in variety R, jasmonic acid content increased and then decreased. We found that salicylic acid has a relatively consistent trend. Notably, *PR-1* homologs were upregulated after 8-d feeding, indicating that salicylic acid regulates the expression of downstream genes. The expression of genes downstream of the jasmonic acid pathway was significantly upregulated after 2-d feeding, suggesting that jasmonic acid pathway genes respond earlier genes of the salicylic acid pathway (Fig. 8).

### Spider mite feeding induced common biotic stress pathways in *C. sinesis*

**Fig. 9:**
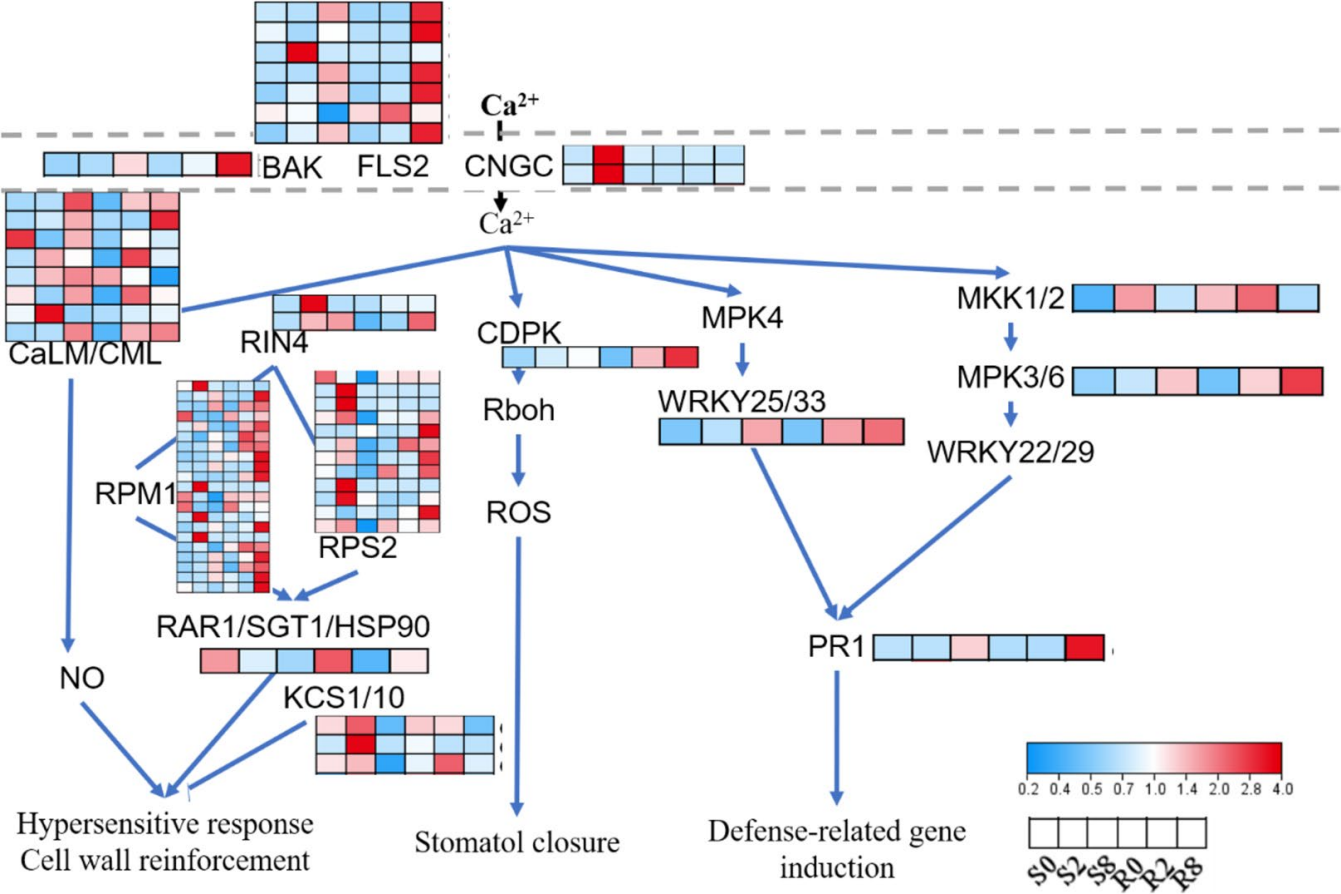
DEGs in plant-pathogen interaction pathways. This figure has obtained the appropriate copyright permission to use the KEGG image. Log2-scaled fragments per kilobase per million (FPKM) values are shown, ranging from low (blue) to high (red) expression. Within each box, rows represented different inoculation time points (from left to right). Black letter mean gene. CDPK, calcium-dependent protein kinase. CaLM, calmodulin. CML, calcium-binding protein. CNGC, cyclic nucleotide-gated ion channel. Rboh, respiratory burst oxidase. MPK3/6, mitogen-activated protein kinase 3/6. MKK1/2, mitogen-activated protein kinase Bruh 1. WRKY22, WRKY transcription factor 22. BAK, brassinosteroid-insensitive 1-associated receptor kinase (1) FLS2, flagellin-sensitive (2) PR1, pathogenesis-related protein 1. RIN4, RPM1-interacting protein 4. RPM1, disease resistance protein RPM1. RPS2, disease resistance protein RPS2. HSP90, heat shock protein 9. KCS1/10, 3-ketoacyl-CoA synthase 1/10

Spider mite feeding may affect the plant–pathogen interaction pathway, with multiple DEGs significantly enriched in this pathway. Expressions of brassinosteroid-insensitive 1-associated receptor kinase 1 (*BAK*) and flagellin-sensitive 2 (*FLS2*) homologs were significantly upregulated at 8 d after spider mite inoculation. By contrast, expression of cyclic nucleotide-gated ion channel (*CNGC*) was rapidly upregulated only in variety S at 8 d after spider mite inoculation. Downstream calcium ions regulated four pathways. Several calcium-binding protein (CML) homologs in the nitric oxide (NO) pathway were upregulated after feeding. However, ChaUn5976.7 (CML23) was upregulated only after spider mite feeding in variety R, and tended to decrease in variety S. NO content is associated with the combined action of CML homologs. In addition, disease resistance protein (RPM1/RPS2)-interacting protein 4 (RIN4) pathway regulates NO. Several RPM1 gene homologs were upregulated in variety R, 8 d after inoculation; however, Cha02g010980, Cha09g010890, Cha12g004730, and Cha14g004360 were significantly upregulated only in variety S 2 d after inoculation. Five *RPS2* homologs were upregulated in variety S 2 d after inoculation, and other *RPS2* homologs were mainly upregulated 8 d after inoculation in variety R. 3-ketoacyl-CoA synthase (KCS) inhibits the hypersensitive response. Three *KCS* homologs were upregulated 2 d after inoculation, of which Cha09g006290 and Cha09g010890 were significantly upregulated in variety S, and Cha02g017330 was significantly upregulated in variety R, suggesting that *KCS* is related to the early stress response. Ca^2+^ activates ROS: calcium-dependent protein kinase (CDPK) was mainly upregulated in variety R after inoculation. We speculate that variety R responds to biotic stress by increasing ROS production. In addition, several genes in the MAPK pathway are involved in regulating *PR-1* expression. Of these, expression of WRKY transcription factor showed upregulation earlier in variety R (Fig. 9).

## Discussion

Tea is a natural and nonpolluting green food. However, several pests threaten the tea plant, with six-spotted spider mites being one of the most persistent and harmful in China. Developing tea varieties naturally-resistant to spider mites would reduce insecticide use. Most studies on tea plants have focused on tea quality and flavor, and few germplasm resources for natural resistance are available. The limited knowledge of genetic resources and resistance molecules in tea significantly hinders development of molecular breeding for resistance in tea. In this study, *Tianfu-5* (resistant strain) was used to compared with *Fuding Dabai* (Susceptible). We integrated transcriptomic and metabolomic methods to identify candidate genes and metabolites produced in resistance to spider mites.

Although we used the same material for transcriptome and metabolome analyses, the number of DEGs in different comparison groups was much greater than the number of DAMs, which may be related to the relatively small number of known metabolites in the library. Interestingly, DEGs of varieties R and S on d 2 and d 8 after spider mite feeding, and DEGs between time periods of variety R, were significantly enriched in pathways including phenylpropanoid biosynthesis, MAPK signaling pathway, plant–pathogen interaction, and alpha-linolenic acid metabolism (Fig. 2E, F). Combined with metabolomic results, DAMs were significantly enriched in phenylpropanoid biosynthesis and phenylalanine metabolism pathways (Fig. 4D, E).

The phenylpropanoid biosynthesis pathway is closely related to the synthesis of the cell wall through regulation of lignin biosynthesis [40, 41]. Increase in secondary cell wall in response to biotic and abiotic stress protects against pests and diseases [40–48]. The spider mite sucks cellular contents from tea leaves, producing tiny, pale spots or scars that induce a biotic stress response. Correlation analysis showed that candidate DEGs and DAMs correlated with phenylpropanoid biosynthesis and flavonoid biosynthesis pathways, and the expression of related genes was mainly upregulated or progressively upregulated on d 2 and d 8 after spider mite feeding, suggesting that the expression of these genes may be induced.

Phytohormones are involved in biotic stress responses, having a direct or indirect role in enhancing plant resistance [49–53]. Salicylic acid, jasmonic acid, and brassinosteroids have been extensively studied in biotic stresses [49–51, 53]. Evaluating the role of a single phytohormone in response to biotic stress is challenging because of significant crosstalk between plant hormones [54]. In this study, four phytohormones were detected in metabolomic data. Of these, salicylic acid and jasmonic acid were significantly different between several comparison groups. Salicylic acid and jasmonic acid signaling triggers plant immunity against sucking and chewing insects, respectively [55]. In varieties S and R, salicylic acid gradually increased after spider mite feeding. Moreover, downstream genes of salicylic acid, including several *PR-1* homologs, were significantly upregulated on d 8 after spider mite feeding, suggesting that salicylic acid signaling responds to biotic stress, but not to differences in variety. However, jasmonic acid signaling significantly decreased in variety S and increased in variety R on d 2, indicating that jasmonic acid signaling responds to differences in resistance between the two varieties. *JAR1* (downstream of jasmonic acid signaling) expression was upregulated in variety R (R2 and R8) compared with variety S (S8) (Fig. 8). In tomato, MYC2 activates gene expression downstream of JA receptors and mediates activation of the wound response [55]. In this study, two MYC2 homologs were upregulated after spider mite feeding. Studies must examine the role of MYC2 in activating JA receptors in tea after spider mite feeding.

Interestingly, the DEGs were significantly enriched in plant–pathogen interaction pathways. To our knowledge, most plant-feeding insects carry pathogens, which infect plants. We speculate that spider mites carry pathogens that infect tea plants to induce the plant–pathogen interaction pathway [56]. In plant–pathogen interaction pathways, calcium ions regulate at least four downstream sub-pathways. *CaLM* and *RIN4* are mainly involved in the regulation of NO and regulate the hypersensitive response and cell wall reinforcement. Because multiple homologous genes may be involved in the regulation of the pathway, their combined effect may differ between the two varieties. Moreover, calcium also regulates ROS production [57]. *CDPK* expression is significantly upregulated in variety R compared with variety S. However, whether ROS is involved in response to spider mite feeding is unclear. In addition, calcium regulates *PR-1* expression through WRKY25/33 or MKK1/2 and MPK3/6 pathways. WRKY transcription factors may play important regulatory roles in spider mite stress response. Consistently, *WRKY33* homologs showed a greater tendency of upregulation in variety R. In plants, the MAPK pathway plays an important role in disease and pest resistance [58]. *MKK1/2* and *MPK3/6* are two most important genes of the MAPK and plant–pathogen interaction pathways [59–61]. *MKK1/2* and *MPK3/6* homologs showed a greater tendency of upregulation in variety R compared with variety S. Thus, induction of ROS and *PR-1* expression through plant–pathogen interaction pathways improves the stress response and resistance of variety R against spider mites.

## Conclusion

This study examined genetic and biochemical changes in tea plant at three time points after spider mite feeding. We screened out candidate DEGs and DAMs in several comparison groups. DEGs and DAMs were enriched in pathways including phenylpropanoid biosynthesis, MAPK signaling, plant–pathogen interaction, alpha-linolenic acid metabolism, salicylic acid and jasmonic acid synthesis, etc. The results suggest that lignin and plant hormone play crucial roles in tea plant resistance against spider mites. Our findings provide insights into plant–insect interactions and will serve as a foundation for studies on improving tea plant resistance against spider mites.

### Supplementary Information

**Additional file 1: Supplementary Figure 1.** The GO enrichment analysis.

**Additional file 2: Supplementary Figure 2.** The KEGG enrichment analysis.

**Additional file 3: Supplementary Figure 3.** The correlation analysis of top250 DEGs and DAMs.

**Additional file 4: Supplementary Table 1.** Data filtering statistics.

**Additional file 5: Supplementary Table 2.** Statistical of base information.

**Additional file 6: Supplementary Table 3.** The statistical of mapping reference genome.

**Additional file 7: Supplementary Table 4.** The mapping region statistics.

**Additional file 8: Supplementary Table 5.** Identification of metabolites.

**Additional file 9: Supplementary Table 6.** The correlation analysis list of DEGs and DAMs.

**Additional file 10: Supplementary Table 7.** The correlation matrix of 23 DAMs and 118 DEGs.

**Additional file 11: Supplementary Table 8.** The WRKY40 transcription factor targets predicted.

**Additional file 12: Supplementary Table 9.** The gene expression patten in phenylpropanoid biosynthesis pathways.

## Data Availability

The RNA-seq datasets are available in the NCBI repository (http://www.ncbi.nlm.nih.gov/bioproject/PRJNA996324).
