# Supplementary figures and images for: Transcriptomic and metabolomic analyses reveals keys genes and metabolic pathways in tea (Camellia sinensis) against six-spotted spider mite (Eotetranychus Sexmaculatus)
Source: BMC Plant Biol. 2023 Dec 11;23:638. doi: 10.1186/s12870-023-04651-8 (PMC10712147; doi:10.1186/s12870-023-04651-8)

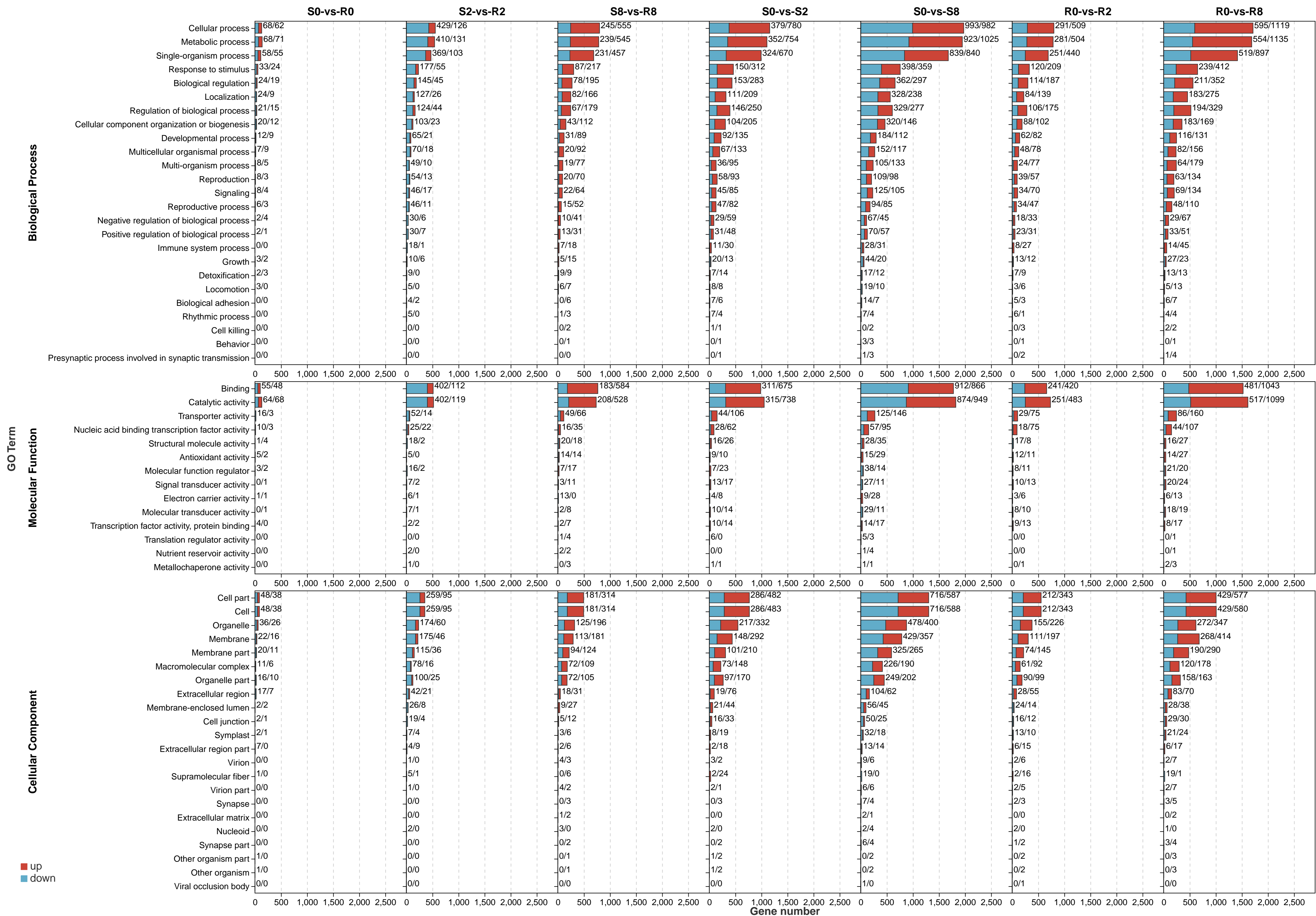

Supplement: Supplementary file 1 — Additional file 1: Supplementary Figure 1. The GO enrichment analysis. [file 12870_2023_4651_MOESM1_ESM.pdf]

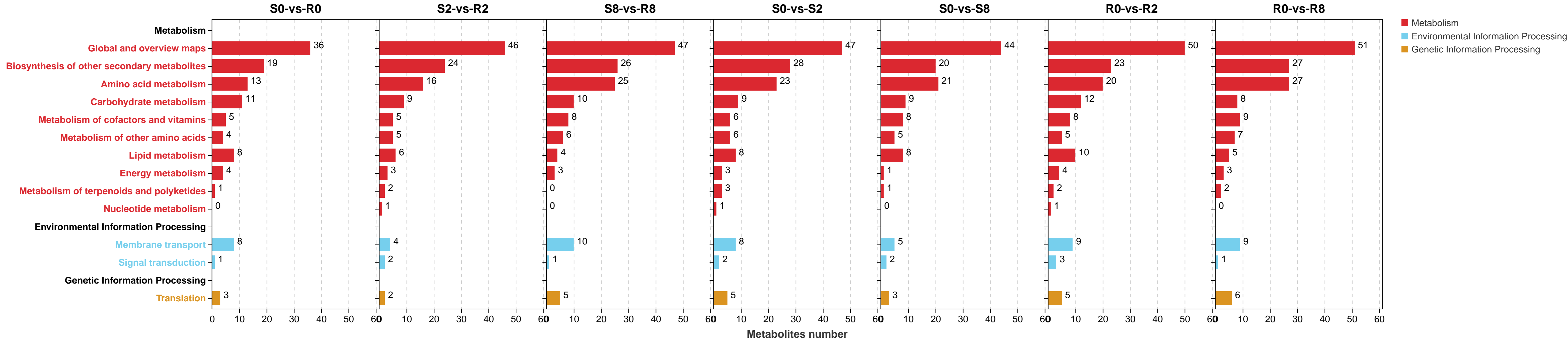

Supplement: Supplementary file 2 — Additional file 2: Supplementary Figure 2. The KEGG enrichment analysis. [file 12870_2023_4651_MOESM2_ESM.pdf]

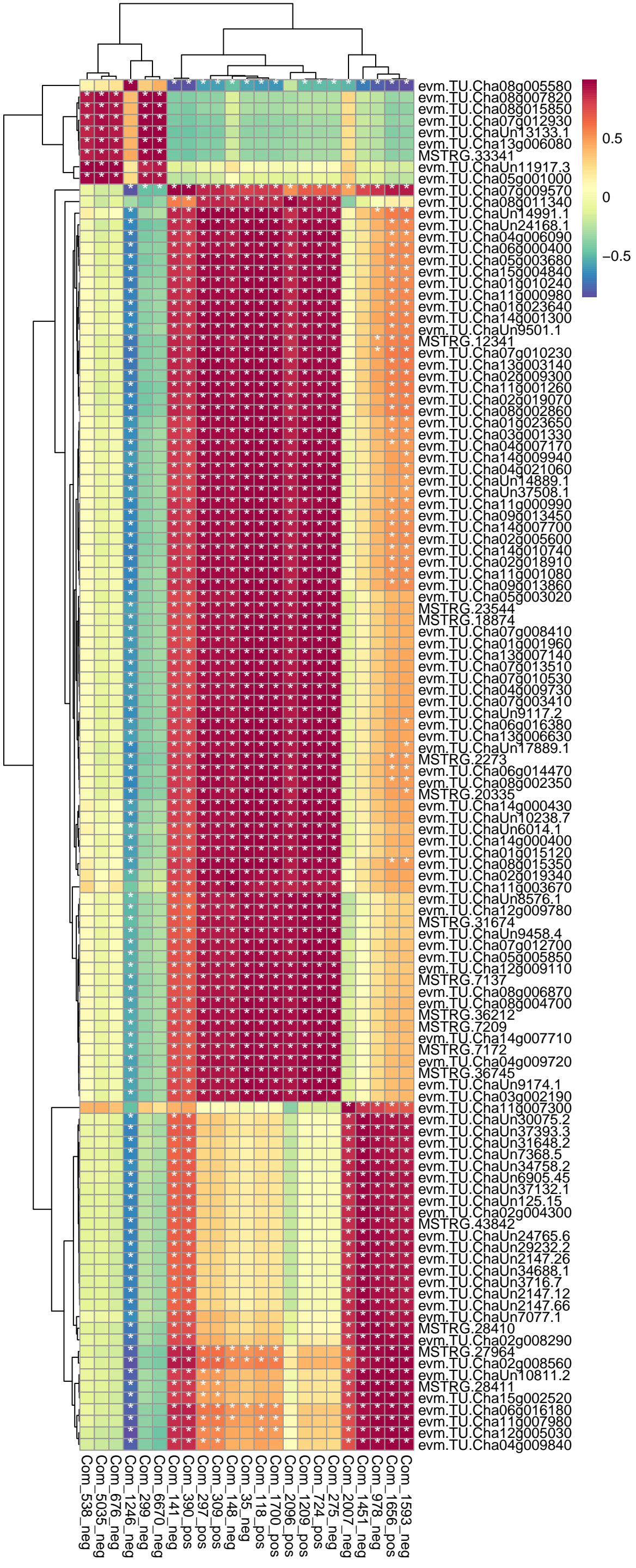

Supplement: Supplementary file 3 — Additional file 3: Supplementary Figure 3. The correlation analysis of top250 DEGs and DAMs. [file 12870_2023_4651_MOESM3_ESM.pdf]
